# Analysis of risk factors for polypharmacy and excessive polypharmacy

**DOI:** 10.3389/fmed.2026.1818441

**Published:** 2026-08-03

**Authors:** Shigeto Mashiko, Yuiko Suzuki, Yurika Hirota, Hatsuho Ejima, Ryo Nonaka, Saki Nakayama, Emiko Kurosawa, Katsutoshi Furukawa

**Affiliations:** Division of Geriatric and Community Medicine, Faculty of Medicine, Tohoku Medical and Pharmaceutical University, Sendai, Japan

**Keywords:** aging, medical institution, medication, older adults, polypharmacy

## Abstract

**Background:**

Polypharmacy is a growing global concern, particularly among older adults with multimorbidity. Fragmented care and consultations across multiple medical institutions may further increase medication burden. This study aimed to identify risk factors for polypharmacy and excessive polypharmacy in an outpatient population.

**Methods:**

This single-center retrospective observational study included 326 outpatients aged ≥ 65 years at Tohoku Medical and Pharmaceutical University Hospital. Polypharmacy and excessive polypharmacy were defined as the use of ≥5 and ≥10 medications, respectively. The association between age and number of medications was evaluated using Spearman’s rank correlation. Differences in the number of medical institutions consulted were assessed with the Wilcoxon rank sum test. Independent risk factors were identified using multiple logistic regression analysis.

**Results:**

The mean age was 74.0 ± 18.3 years. Age showed a significant positive correlation with the number of medications (*r* = 0.39, *p* < 0.001). Patients with polypharmacy or excessive polypharmacy consulted significantly more medical institutions than those without (*p* = 0.0004 and *p* = 0.0011, respectively). Multivariate analysis demonstrated that age, hypertension, gastrointestinal disorders, diabetes, neuropsychiatric disorders and number of medical institutions consulted were independent risk factors for both polypharmacy and excessive polypharmacy.

**Conclusion:**

Aging and chronic disorders were substantially associated with polypharmacy. Although consultation with multiple medical institutions was statistically associated with increased medication burden, the observed effect size was modest, suggesting that fragmented care may contribute only incrementally to polypharmacy in this population. Integrated care strategies and medication review processes may nevertheless help reduce inappropriate polypharmacy and enhance patient safety.

## 1 Introduction

The global proportion of older adults continues to increase as life expectancy rises. The World Health Organization has identified medication safety as a major global priority, emphasizing the risks associated with inappropriate medication use (1). Older adults frequently experience multimorbidity and consult multiple physicians and medical institutions, resulting in increased medication use and the development of polypharmacy. Polypharmacy has become a major public health concern in aging societies worldwide (2). Polypharmacy and excessive polypharmacy were defined as the concurrent use of ≥5 and ≥10 medications, respectively, in accordance with established literature (3, 4). Polypharmacy is associated with adverse drug reactions, drug-drug interactions, falls, hospitalization, cognitive impairment, and mortality. A systematic review by Maher et al. demonstrated a strong association between polypharmacy and adverse clinical outcomes (5). Similarly, Wastesson et al. reported that excessive polypharmacy is increasingly prevalent among older adults (6).

In addition to multimorbidity, fragmented healthcare delivery is associated with polypharmacy. Patients who consult multiple medical institutions may receive prescriptions from different physicians without adequate coordination. Guthrie et al. showed that multimorbidity combined with fragmented care significantly increases medication burden (7). In Japan, universal health coverage allows unrestricted access to medical institutions, enabling patients to consult multiple providers simultaneously, which may increase the risk of duplicate or unnecessary prescriptions (8). Although previous studies have established associations between aging, chronic disease, and polypharmacy, fewer studies have quantitatively evaluated the relationship between the number of medical institutions consulted and medication burden (9). Therefore, this study examined risk factors for polypharmacy and excessive polypharmacy, focusing on age, comorbidities, and fragmented care in a Japanese outpatient population.

## 2 Materials and methods

### 2.1 Study design

This single-center retrospective observational study included 326 outpatients (135 male, 191 female) aged ≥ 65 years who visited Tohoku Medical and Pharmaceutical University Hospital. The mean age of the participants was 74.0 ± 18.3 (mean ± standard deviation) years. The number of prescribed medications and the number of medical institutions consulted were obtained from electronic medical records. Inclusion criteria were age ≥ 65 years and prescription of ≥1 medication. Exclusion criteria were temporary hospital visits and terminal illness due to any disorder. The measurement period was from January to December, 2024.

### 2.2 Definition of “polypharmacy” and “excessive polypharmacy”

Polypharmacy and excessive polypharmacy were defined as the prescription of ≥5 and ≥10 medications, respectively.

### 2.3 Statistical analyses

The association between age and number of medications was evaluated using Spearman’s rank correlation coefficient. The Wilcoxon rank sum test was used to compare the number of medical institutions consulted between patients with and without polypharmacy and between patients with and without excessive polypharmacy. Multiple logistic regression analysis was performed to identify risk factors for polypharmacy and excessive polypharmacy. Statistical analyses were conducted using the Statistical Analysis System version 9.4 (SAS Institute Inc., Cary, NC, USA). Continuous variables were expressed as mean ± standard deviation. Statistical significance was defined as *p* < 0.05.

### 2.4 Statement of ethics

This study adhered to the Declaration of Helsinki and was approved by the Ethics Committees of Tohoku Medical and Pharmaceutical University Hospital. Because of the retrospective design and complete data anonymization, the requirement for informed consent for participation was waived by the ethics committees.

## 3 Results

**FIGURE 1:**
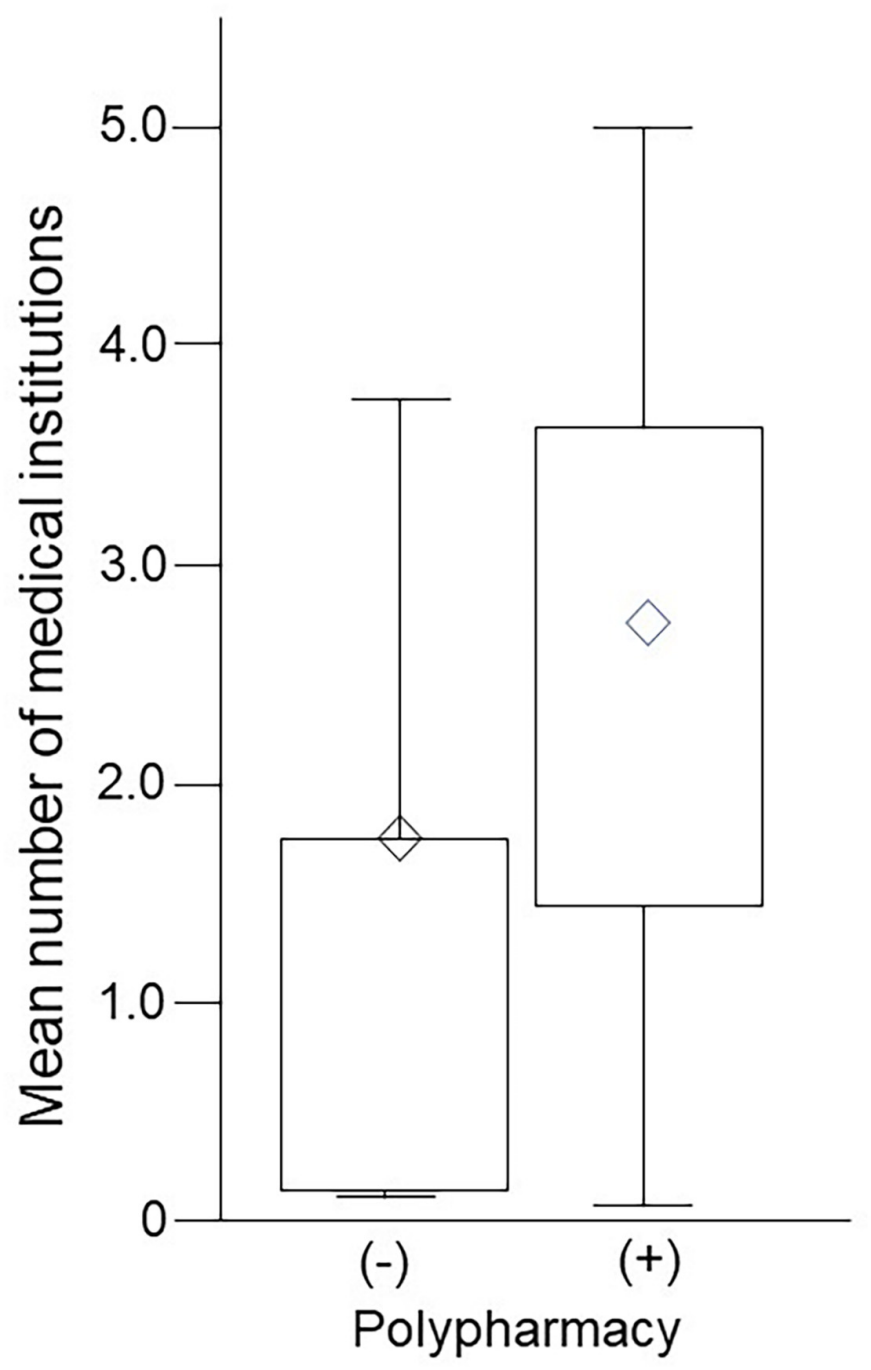
Wilcoxon rank sum test comparing the number of medical institutions consulted between patients with and without polypharmacy.

**FIGURE 2:**
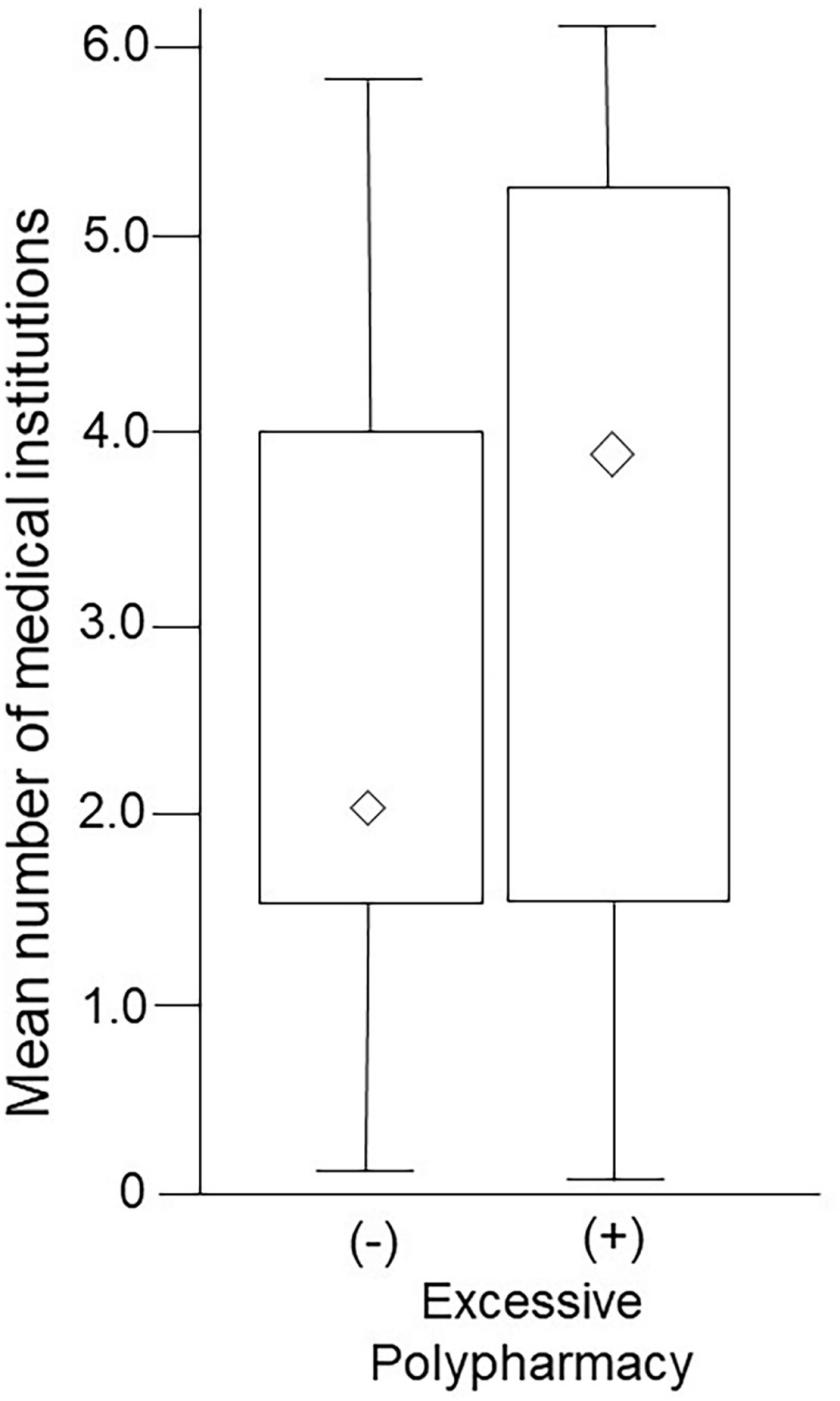
Wilcoxon rank sum test comparing the number of medical institutions consulted between patients with and without excessive polypharmacy.

Table 1 summarizes the demographic characteristics of patients included in this study. As patients were randomly selected from the outpatient clinic, the mean age was 74.0 ± 18.3 years. Comorbidities are presented in Table 1. Hypertension was the most prevalent condition (59.5%), followed by gastrointestinal disorders, including gastroesophageal reflux disease, irritable bowel syndrome, inflammatory bowel disease, gall stones, hepatitis, etc. Spearman’s rank correlation analysis demonstrated a significant positive correlation between age and number of medications (correlation coefficient = 0.39, *p* < 0.001). These findings indicate that medication use increased with advancing age. The Wilcoxon rank sum test showed that patients with polypharmacy consulted significantly more medical institutions than patients without polypharmacy (*p* = 0.0004) (Figure 1). Similarly, patients with excessive polypharmacy consulted significantly more medical institutions than those without excessive polypharmacy (*p* = 0.0011) (Figure 2).

**TABLE 1:** Demographics of participants and their comorbidities.

Demographics	
Age ± SD (years)	74.0 ± 18.3
Sex: male/female	135/191
**Comorbidities**	
Dementia (%)	30.2
Hypertension (%)	59.5
Gastrointestinal diseases (%)	40.5
Dyslipidemia (%)	26.8
Diabetes (%)	38.3
Neuropsychiatric disorders (exc. dementia) (%)	35.2

To elucidate the risk factors [age, dementia, hypertension, gastrointestinal disorders, dyslipidemia, diabetes, neuropsychiatric disorders (including depression, insomnia and anxiety but excluding dementia), and number of medical institutions consulted] that affect polypharmacy, we used multiple logistic regression analysis. Age, hypertension, gastrointestinal disorders, diabetes, neuropsychiatric disorders, and number of medical institutions consulted were significant risk factors for polypharmacy (Table 2). Multiple logistic regression analysis revealed that age, hypertension, gastrointestinal disorders, diabetes, neuropsychiatric disorders, and number of medical institutions consulted were also risk factors for excessive polypharmacy (Table 3).

**TABLE 2:** Multiple logistic regression analysis of risk factors associated with polypharmacy.

	Odds ratio	95% confidence intervals	*P*-value
Age	1.003	1.001 ∼ 1.005	**0.0037**
Dementia	0.4067	0.157 ∼ 1.055	0.0633
Hypertension	8.0534	2.851 ∼ 22.792	**<0.0001**
Gastrointestinal disorders	14.743	5.413 ∼ 40.163	**<0.0001**
Dyslipidemia	1.443	0.5603 ∼ 3.7191	0.4471
Diabetes	15.2	1.894 ∼ 122.25	**0.0106**
Neuropsychiatric disorders (exc. dementia)	4.127	3.203 ∼ 5.050	**0.0026**
Number of medical institutions consulted	1.005	1.0022 ∼ 1.0078	**0.0039**

Bold values indicate statistically significant.

**TABLE 3:** Multiple logistic regression analysis of risk factors associated with excessive polypharmacy.

	Odds ratio	95% confidential interval	*P*-value
Age	1.009	0.984 ∼ 1.036	**0.04775**
Dementia	0.413	0.152 ∼ 1.123	0.07129
Hypertension	4.587	1.974 ∼ 10.665	**0.0004**
Gastrointestinal disorders	8.792	3.253 ∼ 23.96	**<0.0001**
Dyslipidemia	1.728	0.863 ∼ 3.482	0.1276
Diabetes	4.625	2.066 ∼ 10.395	**0.0002**
Neuropsychiatric disorders (exc. dementia)	3.408	1.642 ∼ 7.073	**0.001**
Number of medical institutions consulted	1.0084	1.005 ∼ 1.012	**0.0032**

Bold values indicate statistically significant.

## 4 Discussion

This study demonstrated three key findings. First, age was significantly associated with increased medication use. Second, patients with polypharmacy and excessive polypharmacy consulted significantly more medical institutions. Third, hypertension, gastrointestinal disorders, diabetes, neuropsychiatric disorders, and number of medical institutions consulted were independent risk factors for both polypharmacy and excessive polypharmacy. The association between aging and increased medication use is consistent with previous studies. Multimorbidity increases with age, resulting in guideline-based prescribing. Barnett et al. showed that multimorbidity affects most older adults and is strongly associated with healthcare complexity (10). Fried et al. further emphasized that disease-specific guidelines may unintentionally promote polypharmacy when applied to patients with multiple chronic conditions (11).

Hypertension and diabetes are highly prevalent chronic conditions that often require combination pharmacotherapy according to clinical guidelines (12). Gastrointestinal disorders are also associated due to frequent long-term use of proton pump inhibitors (13). Neuropsychiatric disorders, including insomnia and anxiety, are important contributors, as psychotropic medications are often prescribed long term and may result in prescribing cascades (14). A notable finding of this study was the association between fragmented care and increased medication burden. Patients who consulted multiple medical institutions had significantly higher rates of polypharmacy and excessive polypharmacy. Fragmented care may lead to poor communication between providers, therapeutic duplication, and inadequate medication reconciliation. Athyros et al. highlighted the risks associated with insufficient coordination in patients with multimorbidity (15). Scott et al. emphasized the importance of deprescribing strategies to reduce inappropriate polypharmacy (16). Japan’s free-access healthcare system may facilitate fragmented care, particularly among older adults who consult multiple specialists. Strengthening primary care coordination and implementing systematic medication review may reduce unnecessary prescriptions. Multidisciplinary interventions involving pharmacists, geriatricians, and primary care physicians have demonstrated effectiveness in reducing medication burden and adverse outcomes (17).

This study has several limitations. First, it was conducted at a single center, which may limit the generalizability of the findings to other healthcare settings and populations. Second, although multiple logistic regression analysis was performed, several potentially important confounding factors were not included in the models. In particular, we did not evaluate the overall burden of multimorbidity using validated indices, severity of individual diseases, frailty or functional status, socioeconomic status, or medication appropriateness. These factors are closely associated with both healthcare utilization and prescribing patterns and therefore may have substantially influenced the observed associations. For example, patients with greater multimorbidity or frailty are more likely to require consultations with multiple specialists and simultaneously receive a larger number of medications. Consequently, the association observed between the number of medical institutions consulted and polypharmacy may partly reflect underlying disease complexity rather than fragmented care itself. Similarly, socioeconomic factors may influence healthcare access and consultation behavior, while assessment of medication appropriateness would help distinguish necessary evidence-based polypharmacy from potentially inappropriate prescribing. Because these variables were not available in this retrospective analysis, residual confounding cannot be excluded, and causal interpretations should be avoided. Future multicenter prospective studies incorporating comprehensive measures of multimorbidity, frailty, disease severity, and medication appropriateness are warranted to better clarify the independent contribution of fragmented care to polypharmacy.

In conclusion, aging and chronic diseases were strongly associated with polypharmacy in this outpatient population. Although consultation with multiple medical institutions was independently associated with polypharmacy and excessive polypharmacy, the effect size was modest, and the findings should therefore be interpreted cautiously. Fragmented care may contribute incrementally to medication burden, particularly among patients with complex chronic conditions. Integrated care models, medication reconciliation, and structured deprescribing strategies may help improve medication safety among older adults.

## Data Availability

The datasets used and/or analyzed during the current study are available from the corresponding author on reasonable request.
